# Unlocking new therapeutic horizons through integrative bioinformatics and transcriptomics for drug repositioning in breast cancer therapy

**DOI:** 10.1186/s43046-026-00368-5

**Published:** 2026-05-19

**Authors:** Wirawan Adikusuma, Lalu Muhammad Irham, Rahmat Dani Satria, Ika Nurlaila, Dyah Laksmi Dewi, Firdayani Firdayani, Inna Syafarina

**Affiliations:** 1 Research Center for Computing, Research Organization for Electronics and Informatics, National Research and Innovation Agency, Jakarta, Indonesia; 2 Collaboration Research Center for Precision Oncology Based Omics, Yogyakarta, Indonesia; 3 Faculty of Pharmacy, Universitas Ahmad Dahlan, Yogyakarta, Indonesia; 4 Faculty of Pharmacy, Silpakorn University, Bangkok, Thailand; 5 Department of Clinical Pathology and Laboratory Medicine, Faculty of Medicine, Public Health and Nursing, Gadjah Mada University, Yogyakarta, Indonesia; 6 Integrated Laboratory Installation, Rumah Sakit Umum Pusat Dr. Sardjito, Yogyakarta, Indonesia; 7 Research Center for Vaccine and Drugs, Research Organization for Health, National Research and Innovation Agency, Jakarta, Indonesia; 8 Division of Surgical Oncology, Department of Surgery, Faculty of Medicine, Public Health and Nursing, Gadjah Mada University, Yogyakarta, Indonesia

**Keywords:** Breast cancer, Drug repositioning, Transcriptomics, ITGB7, Vedolizumab

## Abstract

**Background:**

Breast cancer (BRCA) remains one of the most frequently diagnosed malignancies and a leading cause of cancer-related mortality among women worldwide. Its molecular heterogeneity and limited therapeutic options for aggressive subtypes highlight the need for novel treatment strategies. Drug repositioning offers a promising approach by identifying new therapeutic uses for existing drugs with established safety profiles.

**Methods:**

We applied an integrative transcriptomic and bioinformatics framework to identify candidate drug targets and repurposed drugs for BRCA. Differentially expressed genes (DEGs) were identified from four Gene Expression Omnibus (GEO) microarray datasets using the *limma* package with thresholds of |log2 fold change| > 1 and false discovery rate (FDR) < 0.05. Overlapping DEGs were expanded through protein–protein interaction analysis using the STRING database. Functional annotation across ten biological evidence categories was performed using WebGestalt to prioritize BRCA risk genes through a multi-criteria scoring approach. Drug–gene interactions were then analyzed using the Drug–Gene Interaction Database (DGIdb), and tissue-specific gene expression was evaluated using the GTEx database.

**Results:**

Twenty-eight consistently dysregulated genes were identified and expanded into a 77-gene interaction network. Functional prioritization yielded 18 BRCA risk genes, including five druggable targets associated with 11 candidate drugs. ITGB7 emerged as a promising biomarker and therapeutic target, with vedolizumab identified as the top candidate drug.

**Conclusions:**

This study highlights the potential of integrative transcriptomic analysis to identify biomarkers and drug repositioning candidates in BRCA, providing a foundation for further experimental validation.

**Supplementary Information:**

The online version contains supplementary material available at 10.1186/s43046-026-00368-5.

## Introduction

Breast cancer (BRCA) remains the most frequently diagnosed malignancy and a leading cause of cancer-related mortality among women worldwide, representing a substantial global health burden [1]. The disease is clinically and molecularly heterogeneous, comprising multiple subtypes with distinct histopathological characteristics, gene expression profiles, and therapeutic responses [2, 3]. Although early-stage BRCA (non-metastatic) is potentially curable in approximately 70–80% of patients, the overall five-year survival rate is around 91%, indicating that significant challenges remain in disease management [4, 5]. In 2020 alone, an estimated 2.3 million women were newly diagnosed with BRCA, and approximately 685,000 deaths were reported globally [6]. Despite considerable advances in screening, early detection, and therapeutic interventions, recurrence, treatment resistance, and poor prognosis continue to affect patient outcomes, particularly in aggressive subtypes such as triple-negative breast cancer (TNBC) [7]. These challenges highlight the ongoing need to identify novel therapeutic targets and develop more effective treatment strategies.

Drug repositioning has emerged as a promising strategy to address these therapeutic challenges by identifying new therapeutic indications for existing drugs. Compared with de novo drug development, drug repurposing offers several advantages, including reduced development time, lower cost, and improved safety profiles, as many candidate drugs have already undergone clinical testing [8–11]. This approach has been successfully applied in several disease contexts. For example, aspirin, originally developed as an analgesic, has been widely studied for its potential role in the chemoprevention of colorectal cancer [12]. In oncology, drug repositioning provides an opportunity to rapidly explore alternative therapeutic options and identify compounds that may modulate key molecular pathways involved in tumor progression.

Recent advancements in computational biology and omics technologies, particularly transcriptomics, have significantly enhanced the ability to identify candidate drugs for repositioning. Transcriptomic analysis enables the comprehensive profiling of gene expression changes associated with BRCA progression, while integrative bioinformatics tools facilitate the interpretation of these data to identify relevant drug–gene interactions [13]. Several studies have applied transcriptomics-based analyses to identify potential drug candidates by linking disease-associated gene expression signatures with pharmacological databases.

**Fig. 1:**
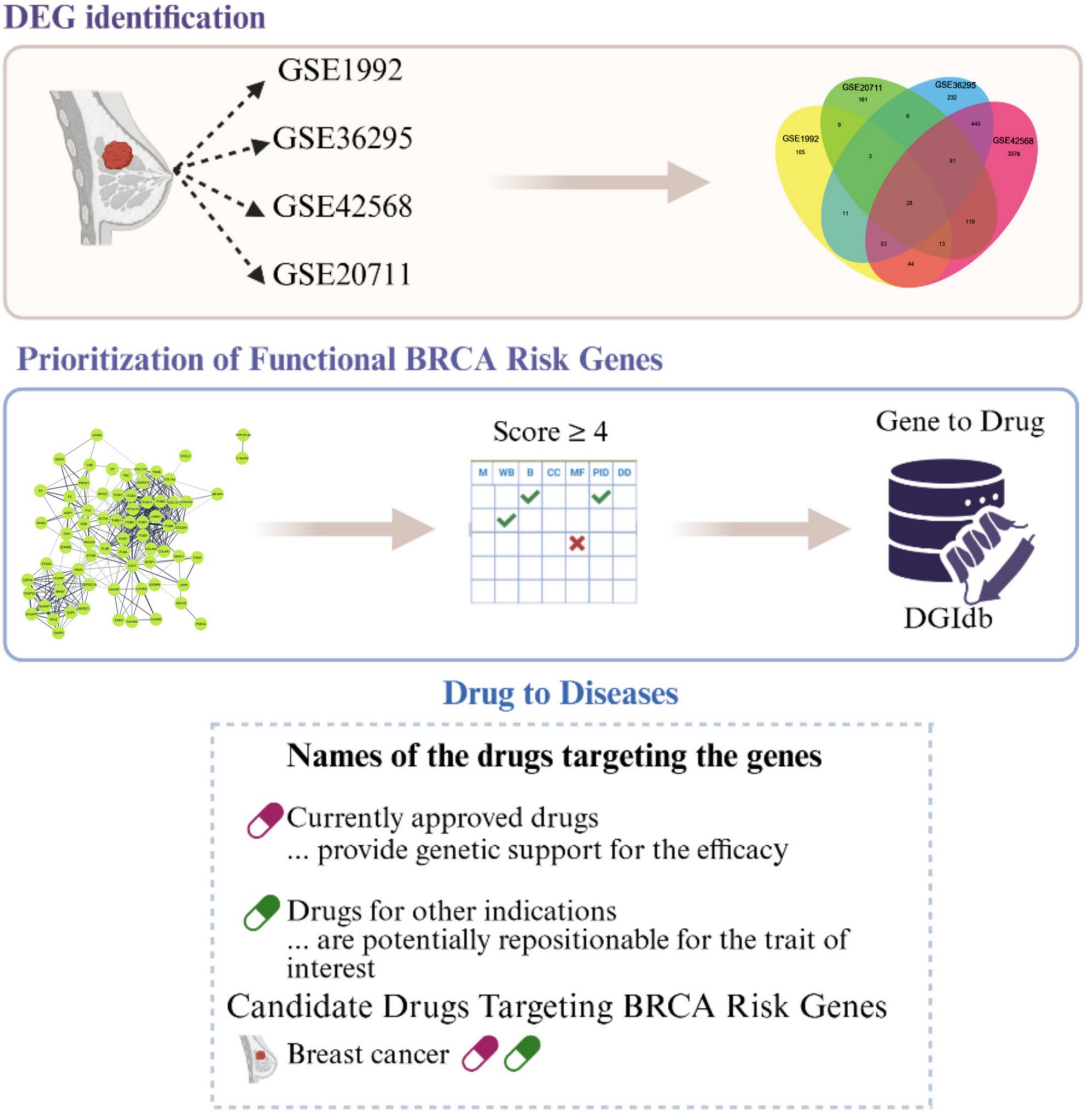
Overview of the integrative bioinformatics workflow used to identify potential drug repositioning candidates in breast cancer (BRCA). The analytical pipeline includes (1) identification of differentially expressed genes (DEGs) from four GEO transcriptomic datasets, (2) intersection analysis to obtain high-confidence DEGs across datasets, (3) protein–protein interaction (PPI) network expansion using STRING, (4) functional annotation and multi-criteria gene prioritization based on biological evidence sources, and (5) drug–gene interaction analysis using the DGIdb database to identify potential repositioning candidates

In this study, we present an integrative bioinformatics framework designed to identify potential drug repositioning candidates for BRCA. Compared with conventional transcriptomics-driven drug repositioning approaches, our framework integrates multi-cohort differential gene expression analysis, protein–protein interaction (PPI) network expansion, and a multi-criteria functional scoring system across ten biological evidence sources to systematically prioritize biologically relevant BRCA-associated genes prior to drug–gene interaction analysis. This layered prioritization strategy aims to enhance biological interpretability and reduce false-positive candidate targets. Using this approach, publicly available gene expression datasets were analyzed to identify differentially expressed genes (DEGs), followed by functional annotation and gene prioritization to define a set of high-confidence biological risk genes. These prioritized genes were subsequently mapped to potential therapeutic agents using the curated Drug–Gene Interaction Database (DGIdb). The overall analytical workflow is illustrated in Fig. 1. This framework provides a systematic strategy for generating hypotheses regarding potential therapeutic targets and repositionable drugs that may warrant further experimental investigation in breast cancer.

## Materials and methods

### Gene expression profile of BRCA

Gene expression microarray datasets related to breast cancer were retrieved from the Gene Expression Omnibus (GEO) database (https://www.ncbi.nlm.nih.gov/geo/, accessed on June 24, 2025), including GSE1992, GSE36295, GSE42568, and GSE20711. Dataset selection followed predefined inclusion criteria: (1) studies involving human breast cancer tissue samples; (2) datasets generated using microarray-based gene expression profiling platforms; (3) availability of normalized or preprocessed expression matrices; and (4) sufficient sample size and metadata to enable comparisons between tumor and non-tumor samples. Datasets were excluded if they involved non-human models, lacked normalization, or used sequencing-based platforms that were not comparable with the selected microarray datasets.

The GSE1992 dataset was generated using the Agilent-012097 Human 1 A Microarray (V2) G4110B platform and focused on identifying intrinsic gene expression signatures of breast tumors [14]. GSE36295 utilized the Affymetrix Human Gene 1.0 ST Array platform, providing transcript-level expression data from breast cancer tissues [15, 16]. Both GSE42568 and GSE20711 were generated using the [HG-U133_Plus_2] Affymetrix Human Genome U133 Plus 2.0 Array, a widely used platform in breast cancer transcriptomic studies [17, 18].

To address cross-platform heterogeneity among GEO datasets, differential expression analysis was performed separately for each dataset using the normalized expression values provided by the original studies. Differentially expressed genes (DEGs) were identified using the limma package in R [19], applying thresholds of |log2 fold change| > 1 and adjusted p-value < 0.05, where adjusted p-values were calculated using the Benjamini–Hochberg method to control the false discovery rate. DEGs that overlapped across the four independent datasets were identified using a Venn diagram to highlight genes consistently dysregulated in BRCA. This multi-cohort intersection strategy was applied to reduce dataset-specific bias and improve the robustness of gene selection for downstream analyses.

### DEG network extension using STRING database

To enhance the identification of potential drug target genes, the initial set of DEGs was expanded through PPI analysis using the STRING database (https://string-db.org, accessed on July 5, 2025). STRING is a comprehensive biological database designed to predict and visualize functional associations between proteins, integrating both direct (physical) and indirect (functional) interactions derived from various sources, including experimental data, computational prediction methods, and public text collections [20]. The curated DEGs from the earlier stages of analysis were submitted to the STRING platform, and a minimum interaction confidence threshold was set to include up to 50 interactions. This step aimed to enlarge the network by incorporating genes functionally linked to the DEGs, thereby increasing the biological context and coverage of the gene set [21]. Expanding the interaction network enhances the likelihood of uncovering novel therapeutic targets, as genes connected through PPI networks often participate in shared biological processes and disease mechanisms. By broadening the DEG landscape through this integrative approach, we aim to capture a more comprehensive repertoire of candidate genes that may be relevant for drug repositioning strategies in BRCA.

### Functional annotation of BRCA risk genes

To identify biologically relevant BRCA risk genes, we performed a functional annotation analysis of DEGs using ten predefined functional categories. These categories included gene ontology terms (biological process, molecular function, cellular component), Kyoto Encyclopedia of Genes and Genomes (KEGG), DisGeNET, Human Cell Landscape, Human Phenotype Ontology, Genome-wide association studies (GWAS), Knockout Mouse Phenotype, and TCGA RNASeq BRCA. Functional enrichment analysis was conducted using the WebGestalt 2024 platform (http://www.webgestalt.org) [22] using the over-representation analysis (ORA) method with a significance threshold of FDR q-value < 0.05.

For gene prioritization, a multi-criteria functional scoring framework was applied. Each gene received one point for significant enrichment within each predefined functional category. The cumulative score for each gene reflects the level of biological support across multiple independent evidence sources. Genes with higher cumulative scores were considered to have stronger biological relevance and were prioritized as biological risk genes for BRCA. The multi-criteria scoring framework was designed to integrate complementary biological evidence from functional annotation, disease association databases, phenotype information, and transcriptomic datasets. Similar evidence-integration strategies have been widely used in genomic network analyses to prioritize biologically relevant disease genes and reduce the likelihood of selecting spurious candidates.

### GTEx-based tissue expression profiling

Tissue-specific gene expression analysis was performed using the Genotype-Tissue Expression (GTEx) Portal (https://gtexportal.org, accessed on July 5, 2025) to assess the expression patterns of candidate genes across a wide range of normal human tissues [23]. The GTEx database provides high-quality RNA sequencing data quantified as transcripts per million (TPM), enabling cross-tissue comparisons of gene expression levels. Each candidate gene identified from the functional prioritization analysis was queried in the GTEx database to evaluate its expression distribution across tissues, including breast tissue and immune-related organs. This analysis provided additional biological context regarding the physiological expression patterns of prioritized genes.

### Prediction of breast cancer drug targets using DGIdb

Potential drug targets for BRCA were identified by analyzing prioritized BRCA risk genes using the Drug–Gene Interaction Database (DGIdb, https://www.dgidb.org/, accessed on July 5, 2025). DGIdb integrates drug–gene interaction data from multiple curated sources and standardizes these interactions into conceptual interaction groups [24]. To identify potential drug repositioning candidates, we focused on FDA-approved drugs with defined interaction types and an interaction score ≥ 1, which reflects the strength and reliability of the reported drug–gene association. This filtering strategy enabled the identification of existing therapeutic compounds that may interact with prioritized BRCA-associated genes and therefore represent potential candidates for drug repositioning.

## Results

### Identification and integration of DEGs across breast cancer datasets

**Fig. 2:**
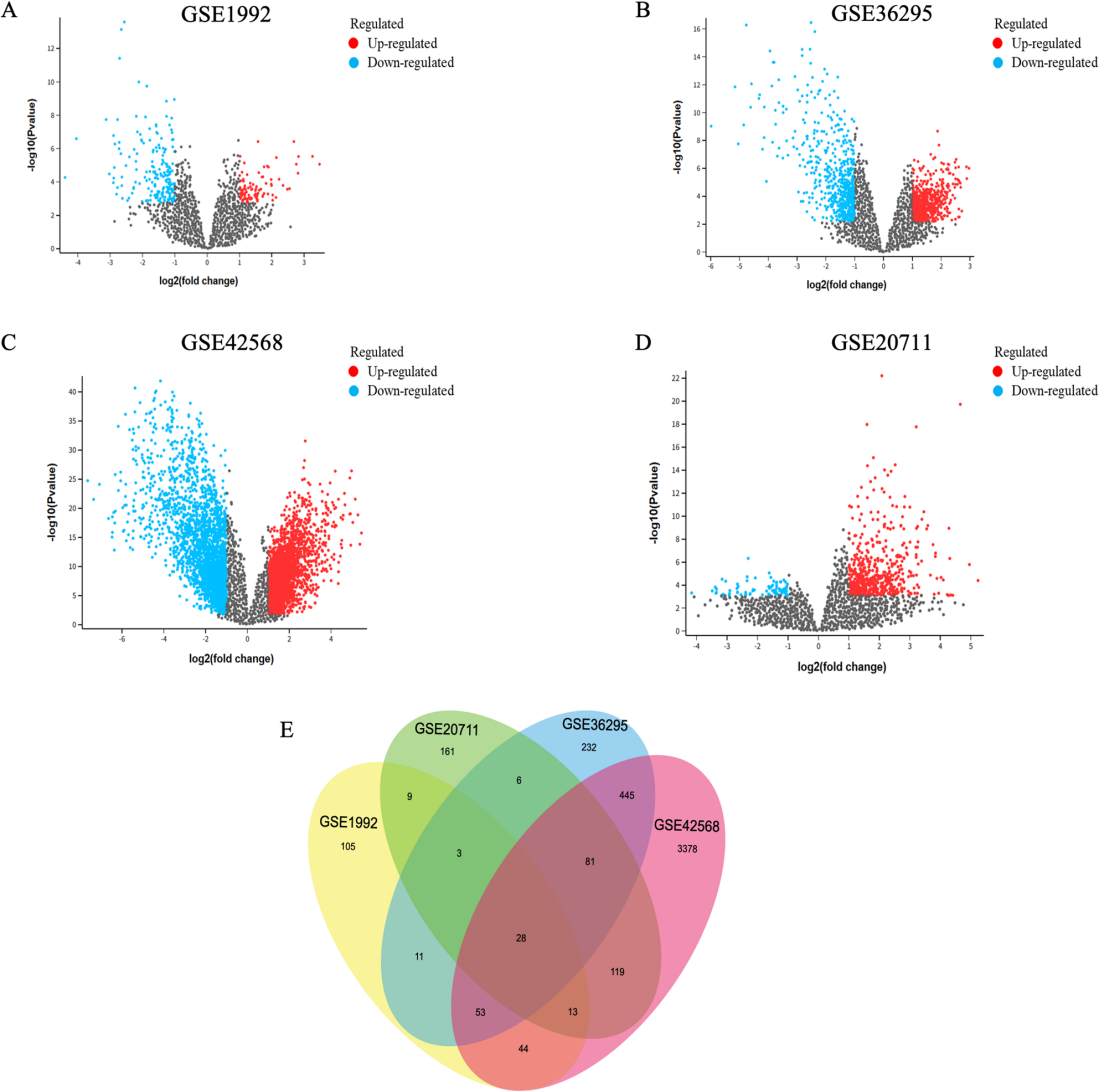
Identification of differentially expressed genes across multiple breast cancer transcriptomic datasets. Volcano plots showing the distribution of upregulated and downregulated genes in four GEO datasets: **A**. GSE1992, **B**. GSE36295, **C**. GSE42568, and **D**. GSE20711. Differential expression analysis was performed using thresholds of **|**log₂ fold change| > 1 and adjusted p-value < 0.05. Upregulated genes are shown in red and downregulated genes in blue. **E**. Venn diagram illustrating the overlap of significant DEGs across the four datasets used to identify high-confidence candidate genes

To identify DEGs associated with BRCA, we analyzed four GEO microarray datasets: GSE1992, GSE36295, GSE42568, and GSE20711, using the limma package in R. DEG selection was based on |log2 fold change| > 1 and adjusted p-value < 0.05. The GSE1992 dataset yielded 277 DEGs (93 upregulated and 184 downregulated), while GSE36295 revealed 924 DEGs (432 upregulated, 492 downregulated). In the GSE42568 dataset, 5720 DEGs were identified (2744 upregulated, 2976 downregulated), and GSE20711 produced 543 DEGs (460 upregulated, 83 downregulated), as illustrated in the volcano plots (Fig. 2A–D). To identify robust and consistently dysregulated genes, we performed an intersection analysis across all four DEG lists. This integrative approach revealed 28 overlapping genes that were differentially expressed across all four datasets (Fig. 2E), representing high-confidence candidates potentially involved in breast cancer pathogenesis. These genes are listed in Supplementary Table S1 and were selected for further enrichment and network-based analyses.

### Prioritization of functional BRCA risk genes via network expansion and scoring system

**Fig. 3:**
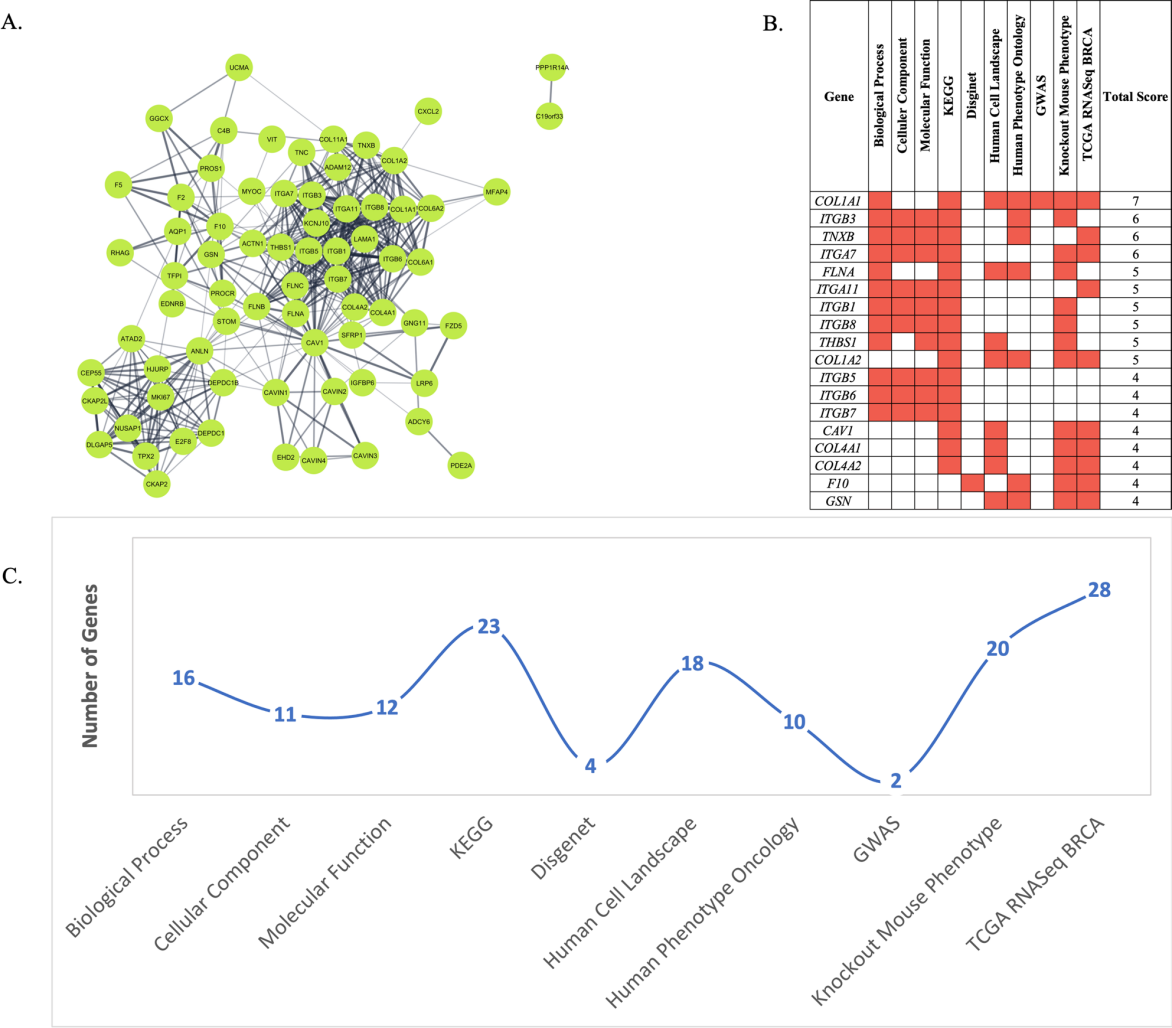
Network-based expansion and functional prioritization of BRCA risk genes. **A**. Protein–protein interaction (PPI) network expansion of 28 high-confidence differentially expressed genes (DEGs) using the STRING database with a minimum interaction confidence score ≥ 0.5, resulting in an expanded network of interconnected genes. **B**. Composite scoring and prioritization of genes based on multi-criteria functional annotation, where each gene receives points based on evidence from multiple biological data sources. **C**. Distribution of gene counts across annotation categories, including Gene Ontology (biological process, molecular function, and cellular component), KEGG pathways, disease associations (DisGeNET), tissue specificity (Human Cell Landscape), phenotype relevance (Human Phenotype Ontology), genome-wide association studies (GWAS), knockout mouse phenotype data, and TCGA BRCA RNA-seq datasets. Functional enrichment analysis was performed using WebGestalt with a significance threshold of FDR q-value < 0.05

To identify biologically relevant BRCA risk genes with therapeutic potential, we conducted a rigorous multi-step functional annotation and network-based prioritization strategy. Initially, 28 high-confidence DEGs—identified from the previous integrative analysis—were subjected to PPI network expansion using the STRING database (version 11.5). To capture additional functionally connected genes, we applied a minimum interaction confidence threshold of 50, resulting in an expanded network comprising 77 genes (Fig. 3A; Supplementary Table S2). This approach allowed us to include indirect yet biologically relevant interactors, increasing the coverage of potentially targetable nodes in the breast cancer molecular landscape.

To systematically prioritize genes for downstream drug discovery, we adopted a composite scoring framework based on functional annotation criteria previously used in previous studies [13, 25–30]. These criteria included enrichment in Gene Ontology categories—biological process (*n* = 16), molecular function (*n* = 11), and cellular component (*n* = 12)—as well as pathway association via KEGG (*n* = 23), disease association from DisGeNET (*n* = 4), tissue-specific expression from the Human Cell Landscape (*n* = 18), phenotype relevance using the Human Phenotype Ontology (*n* = 10), genome-wide association study (GWAS) signals (*n* = 2), functional evidence from Knockout Mouse Phenotype data (*n* = 20), and differential expression from TCGA BRCA RNA-Seq data (*n* = 28). A cumulative scoring system was applied, with higher scores reflecting stronger biological and clinical relevance. As shown in Fig. 3B, genes with a score of ≥ 4 were considered biological BRCA risk genes, yielding 18 prioritized candidates. The distribution of each criterion is shown in Fig. 3C.

### Identification of candidate drugs targeting BRCA risk genes

**Fig. 4:**
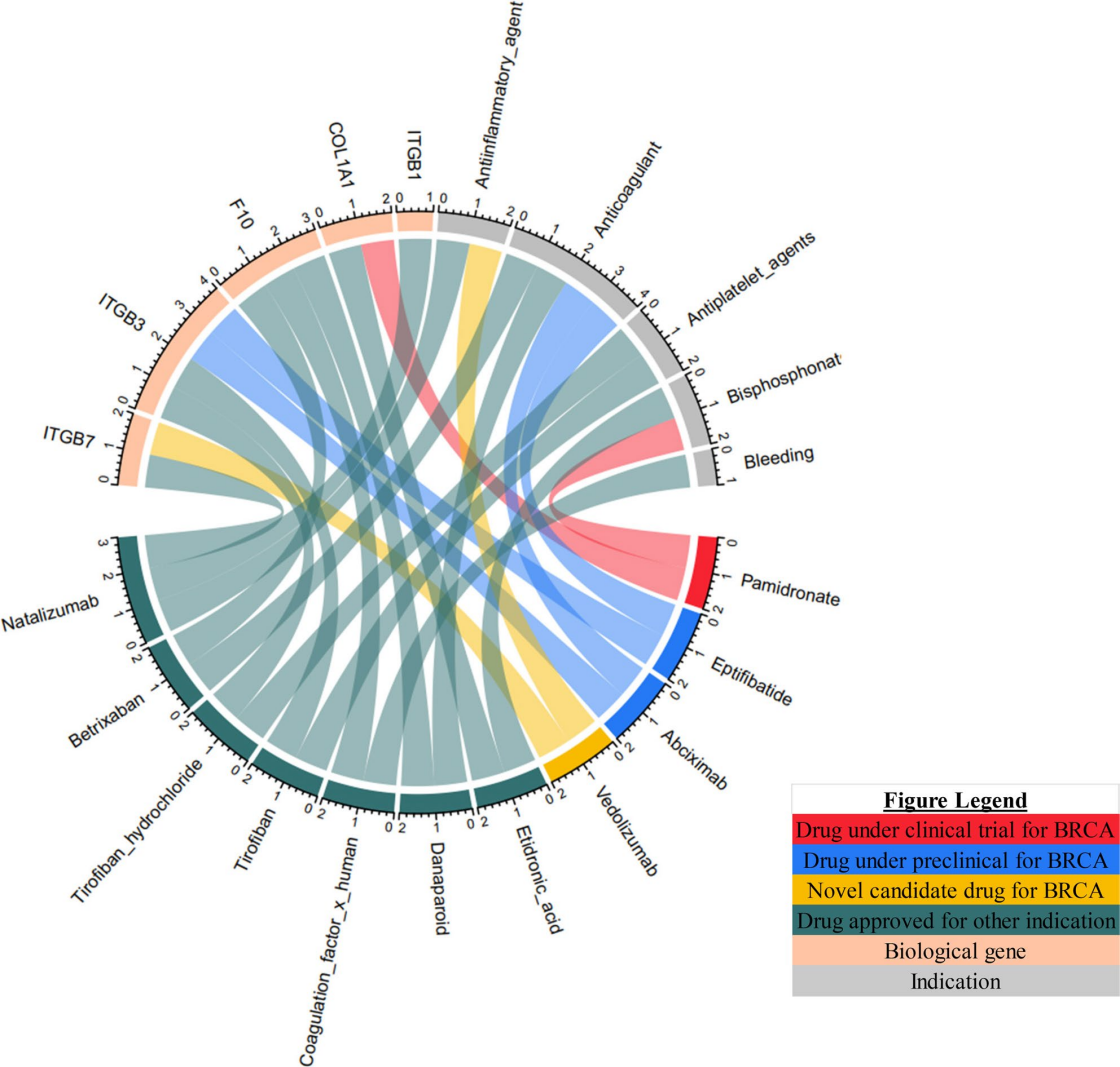
Integrative chord diagram highlighting repurposed drug connections to BRCA risk genes and clinical indications

We mapped 18 biological BRCA risk genes into DGIdb to explore drug–gene interactions. Of these, only 5 genes were druggable, corresponding to 11 drugs (Fig. 4; Supplementary Table S3). Among them, one drug (pamidronate; NCT00128297) is under clinical investigation for BRCA, two drugs (eptifibatide and tirofiban) are in preclinical studies, and 8 have not yet been reported in BRCA therapy. Notably, ITGB7 overlapped with vedolizumab and achieved a high interaction score (8.7), highlighting it as a promising target.

**Fig. 5:**
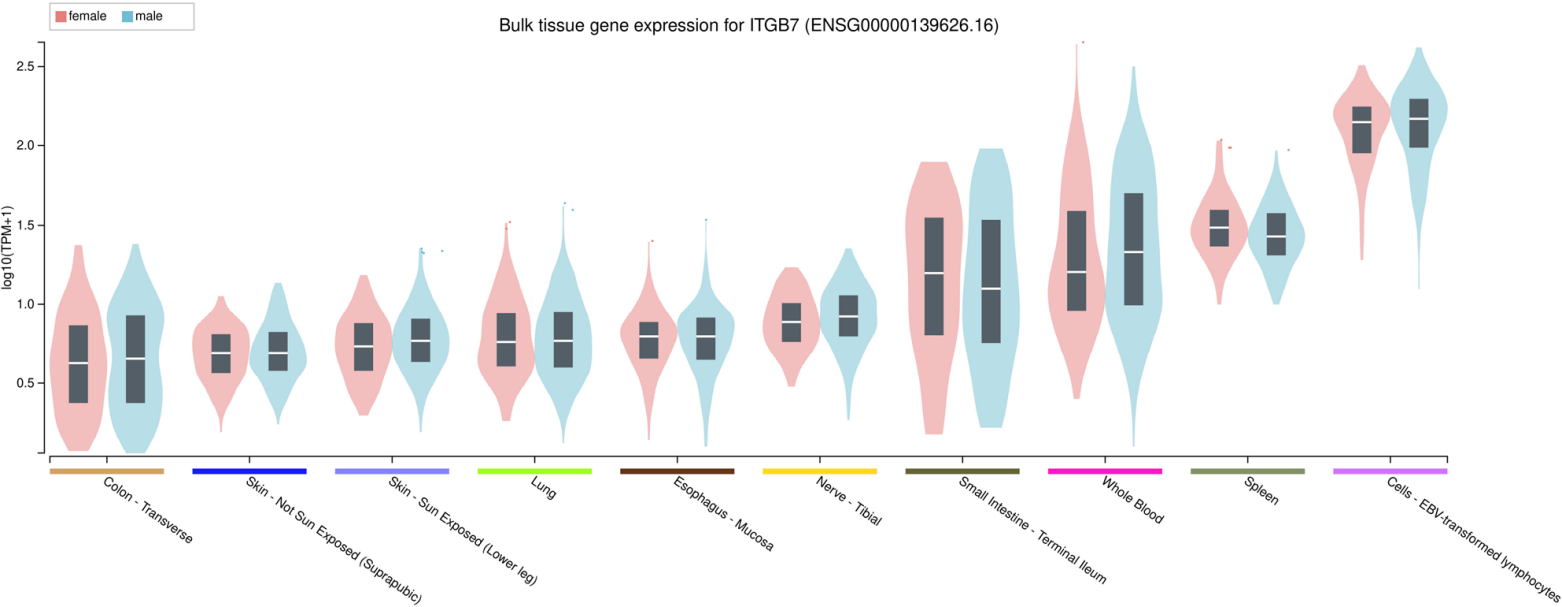
ITGB7 expression patterns across human tissues based on GTEx analysis

To further characterize the biological relevance of ITGB7, we examined its tissue-specific expression using GTEx data (Fig. 5). ITGB7 showed predominant expression in immune-related tissues, including whole blood, spleen, and EBV-transformed lymphocytes, with consistent patterns in both males and females. This profile supports its role in immune modulation and strengthens the rationale for considering ITGB7 as a candidate biomarker in BRCA. Given that ITGB7 is mainly expressed in immune compartments, its dysregulation may influence tumor–immune interactions within the breast cancer microenvironment. This aligns well with the emerging field of immune-oncology, emphasizing the potential impact of this research on advancing breast cancer treatment options.

## Discussion

This study applied an integrative bioinformatics framework combining multi-cohort transcriptomic analysis, PPI network expansion, multi-criteria functional gene prioritization, and drug–gene interaction mapping to identify potential drug repositioning candidates for breast cancer. From the prioritized set of 18 biological risk genes, five were identified as druggable and corresponded to 11 candidate drugs, including pamidronate (currently under clinical investigation), eptifibatide and tirofiban (preclinical), and eight compounds that have not yet been explored in the context of BRCA. Among these candidates, ITGB7 emerged as a particularly notable gene based on its high functional prioritization score and predicted interaction with the therapeutic antibody vedolizumab.

Our findings support the growing role of computational drug repositioning approaches in accelerating early-stage oncology drug discovery. Traditional drug development in breast cancer remains constrained by high cost, lengthy development timelines, and substantial attrition rates, particularly in biologically heterogeneous subtypes such as triple-negative breast cancer (TNBC). Many candidate compounds fail during early clinical phases, highlighting the need for complementary strategies that can systematically identify promising therapeutic targets and compounds with existing safety profiles. In this context, transcriptomics-driven drug repositioning provides an opportunity to leverage disease-associated gene expression signatures to identify candidate drug–gene interactions that may warrant further investigation. Compared with conventional transcriptomics-based approaches, our framework incorporates multi-cohort DEG intersection, PPI-based network expansion, and a multi-criteria functional scoring system integrating ten biological evidence sources.

ITGB7 encodes an integrin β subunit involved in cell–cell adhesion, immune cell trafficking, and signal transduction within the tissue microenvironment. Analysis of GTEx data confirmed that ITGB7 is predominantly expressed in immune-related tissues such as whole blood and spleen, which is consistent with its established role in immune cell homing and lymphocyte migration. Integrins are widely recognized as key regulators of cell adhesion and immune signaling pathways, and their dysregulation has been implicated in tumor progression and tumor–immune interactions across multiple cancer types. Previous studies have suggested that ITGB7 expression may be altered in breast cancer and associated with pathways such as focal adhesion, extracellular matrix (ECM)–receptor interaction, and PI3K–Akt signaling, which play central roles in tumor adhesion, migration, and survival [31].

Emerging evidence also indicates that integrin-mediated signaling pathways may influence tumor metabolism and immune regulation within the tumor microenvironment. For example, recent analyses have suggested that ITGB7 may contribute to metabolic reprogramming and tumor aggressiveness in TNBC through activation of integrin-associated signaling pathways, including FAK and HIF-1α [32]. In addition, computational analyses have reported correlations between ITGB7 expression and immune cell infiltration patterns, including T cells, regulatory T cells, and macrophages, as well as immune exhaustion markers such as CTLA4, LAG3, and TIM-3 [32]. While these findings remain largely correlative, they suggest that ITGB7 may participate in immune-related processes within the BRCA microenvironment and therefore represent a potential candidate biomarker for further investigation.

The identification of vedolizumab, a monoclonal antibody targeting the α4β7 integrin complex, further illustrates the potential of our framework to generate hypotheses regarding drug–gene interactions. Vedolizumab is currently approved for the treatment of inflammatory bowel diseases, where it acts by modulating lymphocyte trafficking to intestinal tissues [33, 34]. Previous studies have demonstrated that β7 integrins play roles in T-cell migration, regulatory T-cell homing, and tissue-resident immune cell populations [35, 36]. In addition, integrin-mediated immune cell interactions have been implicated in antitumor immunity in several cancer models [37]. These observations provide a possible biological rationale for exploring whether modulation of ITGB7-related pathways may influence immune dynamics in the BRCA microenvironment. However, given that our findings are derived entirely from in silico analyses, the proposed relevance of vedolizumab in BRCA should be considered hypothesis-generating and requires further validation in experimental and preclinical BRCA models.

This study has several limitations. First, the transcriptomic datasets analyzed were relatively small and heterogeneous, which may limit statistical power and generalizability despite the use of cross-cohort integration. Second, the drug–gene interactions were derived from the DGIdb, a curated but heterogeneous resource that aggregates evidence from multiple sources. While useful for hypothesis generation, such database-derived interactions do not necessarily indicate direct therapeutic efficacy in a specific disease context and therefore require biochemical and functional validation to confirm binding specificity and pharmacological relevance. In addition, the associations identified between ITGB7 expression, immune infiltration, and metabolic signatures are correlative in nature and cannot establish causality. Reported clinical correlations of ITGB7 in BRCA also remain inconsistent across studies and should be validated in larger and well-annotated patient cohorts. Finally, as this work is based entirely on in silico analyses, further experimental studies, including in vitro functional assays and in vivo preclinical models, are required to substantiate ITGB7 as a therapeutic target and to evaluate the translational potential of candidate drugs such as vedolizumab. Therefore, the candidate drugs identified here should be considered starting points for future translational investigations rather than immediate therapeutic strategies for BRCA treatment.

## Conclusion

In summary, our integrative analysis highlights ITGB7 as a promising biomarker and therapeutic entry point in BRCA, connecting tumor metabolism, immune regulation, and disease progression. The strong interaction with vedolizumab illustrates the potential of drug repurposing strategies to accelerate therapeutic discovery. While experimental validation is still required, these findings provide a compelling rationale for exploring ITGB7-directed therapies to improve outcomes in BRCA. While these findings highlight a potential immune-related therapeutic avenue, they should be interpreted as preliminary computational predictions that require further experimental and translational validation.

## Supplementary Information

Supplementary Material 1. Table S1: Twenty-eight DEGs were collected based on the intersection between GSE1992, GSE36295, GSE42568, and GSE20711.

Supplementary Material 2. Table S2: BRCA-related DEGs expanding by STRING database.

Supplementary Material 3. Table S3: A list of candidate drugs for BRCA based on DGIdb.

## Data Availability

Data is provided within the manuscript or supplementary information files.
